# Stroke Prevention in Atrial Fibrillation: A Scientific Statement of *JACC: Asia* (Part 2)

**DOI:** 10.1016/j.jacasi.2022.06.004

**Published:** 2022-08-23

**Authors:** Chern-En Chiang, Tze-Fan Chao, Eue-Keun Choi, Toon Wei Lim, Rungroj Krittayaphong, Mingfang Li, Minglong Chen, Yutao Guo, Ken Okumura, Gregory Y.H. Lip

**Affiliations:** a General Clinical Research Center, Taipei Veterans General Hospital, Taipei, Taiwan; b Division of Cardiology, Department of Medicine, Taipei Veterans General Hospital, Taipei, Taiwan; c School of Medicine, National Yang Ming Chiao Tung University, Taipei, Taiwan; d Institute of Clinical Medicine, and Cardiovascular Research Center, National Yang Ming Chiao Tung University, Taipei, Taiwan; e Department of Internal Medicine, Seoul National University Hospital, Seoul, Republic of Korea; f National University Heart Centre, National University Hospital, Singapore, Singapore; g Division of Cardiology, Department of Medicine, Faculty of Medicine Siriraj Hospital, Mahidol University, Bangkok, Thailand; h Division of Cardiology, First Affiliated Hospital of Nanjing Medical University, Nanjing, China; i Department of Pulmonary Vessel and Thrombotic Disease, Sixth Medical Centre, Chinese PLA General Hospital, Beijing, China; j Liverpool Centre for Cardiovascular Science, University of Liverpool & Liverpool Heart and Chest Hospital, Liverpool, United Kingdom; k Division of Cardiology, Saiseikai Kumamoto Hospital, Kumamoto, Japan; l Department of Clinical Medicine, Aalborg University, Aalborg, Denmark

**Keywords:** Asia, atrial fibrillation, NOAC, stroke, VKA, AF, atrial fibrillation, CKD, chronic kidney disease, CrCl, creatinine clearance, eGFR, estimated glomerular filtration rate, ESRD, end-stage renal disease, LAA, left atrial appendage, NOAC, non-vitamin K antagonist oral anticoagulant, OAC, oral anticoagulant, VKA, vitamin K antagonist

## Abstract

Atrial fibrillation (AF) is the most common sustained cardiac arrhythmia and is associated with substantial increases in the risk for stroke and systemic thromboembolism. With the successful introduction of the first non-vitamin K antagonistdirect oral anticoagulant agent (NOAC) in 2009, the role of vitamin K antagonists has been replaced in most clinical settings except in a few conditions for which NOACs are contraindicated. Data for the use of NOACs in different clinical scenarios have been accumulating in the past decade, and a more sophisticated strategy for patients with AF is now warranted. *JACC: Asia* recently appointed a working group to summarize the most updated information regarding stroke prevention in AF. The aim of this statement is to provide possible treatment options in daily practice. Local availability, cost, and patient comorbidities should also be considered. Final decisions may still need to be individualized and based on clinicians’ discretion. This is part 2 of the statement.

## NOACs in Patients With Chronic Kidney Disease

Patients with atrial fibrillation (AF) and chronic kidney disease (CKD) have increased risk for thromboembolism and bleeding events.^1^ The global burden of CKD is substantial and growing.^2^ Both incidence rates and prevalence rates of treated end-stage renal disease (ESRD) rose significantly from 2003 to 2016 in East Asian and Southeast Asian countries.^3^ In fact, Taiwan and Japan reported the highest prevalence rates of ESRD in the world,^3^ while countries with the highest percentage increases in ESRD prevalence were Taiwan, the United States, South Korea, and Thailand.^3^ As renal function is a key factor in determining doses of non-vitamin K antagonist oral anticoagulant agents (NOACs), Asian patients more commonly received reduced doses of NOACs in clinical trials. Table 1 shows the differences in the 3 key factors in dose selection (renal function, age, and body weight) among Asians vs non-Asians in the 4 NOAC trials.^4^, ^5^, ^6^, ^7^ In the ENGAGE-AF (Effective Anticoagulation With Factor Xa Next Generation in Atrial Fibrillation) trial, 46.9% of Asians received reduced doses, compared with only 23.2% of non-Asians.

**Table 1:** Renal Function, Age, and Body Weight Among Asians vs Non-Asians

	RE-LY^4^		ROCKET AF^5^		ARISTOTLE^6^		ENGAGE-AF^7^	
	Asians (n = 2,782)	Non-Asians (n = 15,331)	Asians (n = 932)	Non-Asians (n = 13,322)	Asians (n = 1,993)	Non-Asians (n = 16,208)	Asians (n = 1,943)	Non-Asians (n = 19,162)
CrCl <50 mL/min	26.6%	18.4%	65 mL/min	73 mL/min	23.1%	15.8%	30%	18.2%
Median age, y	68	72	70	71	69	70	70	71
Median weight, kg	66	86	67	83	67	84	67	86
Reduced dose	NA^a^	NA^a^	NR	NR	4.77%	4.34%	46.9%	23.2%

ARISTOTLE = Apixaban for Reduction in Stroke and Other Thromboembolic Events in Atrial Fibrillation; CrCl = creatinine clearance; ENGAGE-AF = Effective Anticoagulation With Factor Xa Next Generation in Atrial Fibrillation; NA = not available; NR = not reported; RE-LY = Randomized Evaluation of Long Term Anticoagulant Therapy; ROCKET AF = Rivaroxaban Once Daily Oral Direct Factor Xa Inhibition Compared with Vitamin K Antagonism for Prevention of Stroke and Embolism Trial in Atrial Fibrillation.

a There were no dose reduction criteria in the RE-LY trial.^8^

Three major trials of NOACs excluded patients with severe CKD (creatinine clearance [CrCl] 15-29 mL/min) and patients with ESRD (CrCl <15 mL/min and/or dialysis).^8^, ^9^, ^10^ The ARISTOTLE (Apixaban for Reduction in Stroke and Other Thromboembolic Events in Atrial Fibrillation) trial is the only one that extended enrollment to patients with CrCl ≥25 mL/min.^11^ The predefined subgroup analyses of the efficacy and safety of NOACs vs warfarin in patients with different renal function (CrCl <50, 50 to <80, and ≥80 mL/min) are shown in Table 2. In general, efficacy and safety were consistent in different ranges of renal function. Apixaban performed better on safety endpoints in patients with CrCl <50 mL/min, probably because it has the lowest percentage of renal clearance. Subanalyses in Asian patients by renal function were available from the RE-LY (Randomized Evaluation of Long Term Anticoagulant Therapy) trial^12^ and the J-ROCKET AF (Japan-Rivaroxaban Once Daily Oral Direct Factor Xa Inhibition Compared with Vitamin K Antagonism for Prevention of Stroke and Embolism Trial in Atrial Fibrillation) trial^13^ (Table 2). Both efficacy and safety in Asians are consistent in different ranges of renal function and show similar pattern as global data.

**Table 2:** Subgroup Analyses of NOAC Effects Based on Renal Function in Clinical Trials

	Efficacy (Stroke/SEE)			*P*_interaction_	Safety (Major Bleeding)			*P*_interaction_
Global data								
CrCl, mL/min^a^	≥80	50-<80	<50		≥80	50-<80	<50	
Dabigatran 110 mg^15^	0.84 (0.54-1.32)	0.93 (0.70-1.23)	0.85 (0.59-1.24)	0.9108	0.61 (0.44-0.84)	0.76 (0.62-0.94)	0.99 (0.77-1.28)	0.0607
Dabigatran 150 mg^15^	0.67 (0.42-1.09)	0.68 (0.50-0.92)	0.56 (0.37-0.85)	0.7522	0.84 (0.62-1.13)	0.91 (0.75-1.11)	1.01 (0.79-1.30)	0.6393
Rivaroxaban 20 mg^9^	0.94 (0.67-1.31)	0.85 (0.67-1.08)	0.88 (0.65-1.19)	0.900	1.06^b^ (0.92-1.21)	1.04^b^ (0.93-1.15)	0.98^b^ (0.84-1.14)	0.735
Apixaban^16^	0.88 (0.64-1.22)	0.74 (0.56-0.97)	0.79 (0.55-1.14)	0.705	0.80 (0.61-1.04)	0.77 (0.62-0.94)	0.50 (0.38-0.66)	0.03
CrCl, mL/min^a^	>95	50-95	<50		>95	50-95	<50	
Edoxaban 60 mg^17^	1.36 (0.88-2.10)	0.78 (0.64-0.96)	0.87 (0.65-1.18)	0.08	0.60 (0.42-0.85)	0.89 (0.75-1.04)	0.76 (0.58-0.98)	0.11
Asian data								
CrCl, mL/min^a^	≥80	50-<80	<50		≥80	50-<80	<50	
Dabigatran 110 mg^12^	0.43 (0.11-1.65)	0.83 (0.48-1.44)	0.97 (0.50-1.88)	0.56	0.20 (0.02-1.68)	0.54 (0.31-0.94)	0.62 (0.35-1.11)	0.60
Dabigatran 150 mg^12^	0.41 (0.11-1.60)	0.36 (0.18-0.73)	0.61 (0.28-1.29)	0.62	0.98 (0.28-3.38)	0.49 (0.27-0.86)	0.55 (0.30-1.01)	0.62
CrCl, mL/min	≥50		<50		≥50		<50	
Rivaroxaban 15 mg (J-ROCKET AF)^13^	0.36 (0.14-0.93)		0.82 (0.25-2.69)	0.279	1.07^b^ (0.80-1.43)		1.22^b^ (0.78-1.91)	0.628

Values are HR (95% CI) unless otherwise indicated.

CrCl = creatinine clearance; J-ROCKET AF = Japan-Rivaroxaban Once Daily Oral Direct Factor Xa Inhibition Compared with Vitamin K Antagonism for Prevention of Stroke and Embolism Trial in Atrial Fibrillation; SEE = systemic embolization event.

a Calculated using the Cockcroft-Gault equation.^14^

b Major and nonmajor clinically relevant bleeding.

Renal function should be regularly evaluated by calculating CrCl using the Cockcroft-Gault equation,^14^ as changes in renal function are related to the adjustment of NOAC doses and can also have an impact on the risk for bleeding and stroke.

### Patients with mild to moderate CKD (CrCl 30-49 mL/min)

Compared with warfarin, 4 NOACs showed consistent efficacy and safety in subgroup analyses of patients with CrCl of 30 to 49 mL/min in pivotal trials^9^^,^^12^^,^^13^^,^^15^, ^16^, ^17^ (Table 2). Meta-analyses also indicated that NOACs were superior to warfarin in preventing thromboembolic events and lowering the risk for bleeding in individuals with AF and mild to moderate CKD.^18^^,^^19^ In several large observational studies based on Asian populations, all 4 NOACs also showed comparable or lower risk for thromboembolism and a lower risk for bleeding than warfarin in patients with mild to moderate CKD.^20^^,^^21^

### Patients with severe CKD (CrCl 15-29 mL/min)

Major NOAC trials, except the ARISTOTLE trial, excluded patients with CrCl < 30 mL/min. Dabigatran should not be used in patients with severe CKD, as its renal clearance is about 80%. In a subanalysis of patients with advanced CKD (CrCl 25-30 mL/min) in the ARISTOTLE trial, apixaban caused less major bleeding (HR: 0.34; 95% CI: 0.14-0.80), less major or clinically relevant nonmajor bleeding (HR: 0.35; 95% CI: 0.17-0.72), and a numerically lower risk for stroke or systemic embolization (HR: 0.55; 95% CI: 0.20-1.51) compared with warfarin.^22^ In the ELDERCARE-AF (Edoxaban Low-Dose for Elder Care Atrial Fibrillation Patients) trial, which included 41% of elderly patients with CrCl of 15 to 30 mL/min, edoxaban 15 mg showed a reduced risk for primary composite efficacy endpoints (stroke, systemic embolization, and cardiovascular death) (HR: 0.34; 95% CI: 0.19-0.61; *P* < 0.001) and a nonsignificant increase in major bleeding (HR: 1.87; 95% CI: 0.90-3.89; *P* = 0.09).^23^ Accordingly, either apixaban or edoxaban 15 mg may be preferable in these patients.

### Patients with ESRD (CrCl <15 mL/min and/or dialysis)

Patients on dialysis have a higher prevalence of AF and other stroke risk factors, in addition to a higher risk for bleeding. Data for warfarin are controversial. In a retrospective analysis, warfarin decreased cardiovascular events without increasing bleeding.^24^ However, meta-analyses showed that warfarin did not reduce mortality, ischemic events, or stroke and instead increased the risk for significant bleeding.^25^^,^^26^

Use of NOACs in individuals on dialysis is an open question. Meta-analyses of patients with AF on dialysis indicated no benefit in the risk for stroke or systemic thromboembolism but an increased bleeding risk from warfarin, rivaroxaban, and dabigatran compared with no anticoagulant agent or apixaban.^27^^,^^28^ A recent investigation of apixaban showed similar thromboembolic events but lower bleeding risk than with vitamin K antagonists (VKAs).^29^ In another study based on data from U.S. Renal Data System, apixaban was associated with a higher risk for bleeding without a reduction in stroke or systemic thromboembolism compared with no anticoagulation.^30^ So the applicability of apixaban in patients with ESRD is still questionable.^30^ Dabigatran and rivaroxaban increased the risk for hospitalization or death due to bleeding compared with warfarin.^31^ On the basis of this controversial observational evidence, the need for anticoagulation and the choice of NOAC or VKA remains to be determined. Table 3 shows the recommendations for NOAC according to renal function, on the basis of randomized controlled trials.

**Table 3:** Recommended Doses of NOACs by Renal Function According to Randomized Controlled Trials

	Creatinine Clearance, mL/min			
	≥50	30-49	15-29	<15 or Dialysis
Dabigatran	150 mg twice daily^a^	110 mg twice daily	Contraindicated	Contraindicated
Rivaroxaban	20 mg once daily	15 mg once daily	Lack of data	Lack of data
Apixaban	5 mg twice daily^b^	5 mg twice daily^b^	2.5 mg twice daily	Lack of data
Edoxaban	60 mg once daily^c^	30 mg once daily	15 mg once daily	Lack of data

CrCL = creatinine clearance; NOAC = non-vitamin K antagonist oral anticoagulan.

a Dose reduction criterion: 110 mg twice daily if age ≥80 years.

b Dose reduction criteria: 2.5 mg twice daily if ≥2 of the following: age ≥80 years, body weight ≤60 kg, and serum creatinine ≥1.5 mg/dL.

c Dose reduction criteria: 30 mg once daily if ≥1 of the followings: body weight ≤60 kg, CrCl 30 to 50 mL/min, and concomitant use of a potent P-glycoprotein inhibitor.

Recently, 2 clinical trials compared apixaban with VKAs in patients with AF with ESRD (RENAL-AF [Renal Hemodialysis Patients Allocated Apixaban vs. Warfarin in Atrial Fibrillation; NCT02942407] and AXADIA-AFNET 8 [A Safety Study Assessing Oral Anticoagulation With Apixaban vs. Vitamin-K Antagonists in Patients With Atrial Fibrillation and End-Stage Kidney Disease on Chronic Hemodialysis Compare Apixaban and Vitamin-K Antagonists in Patients With Atrial Fibrillation and End-Stage Kidney Disease-Atrial Fibrillation Network 8; NCT02933697]). However, both trials lacked a third treatment arm and encountered significant enrollment difficulties. In fact, the RENAL-AF study was halted prematurely, reporting a numerical doubling of cardiovascular mortality in the apixaban arm compared with the warfarin arm (presented at the 2019 of the American Heart Association).^32^ No conclusion or recommendation could be made from the RENAL-AF study.

For patients with supranormal estimated glomerular filtration rate (eGFR) (>95 mL/min), the U.S. Food and Drug Administration recommended that edoxaban should not be used.^17^ This recommendation was based on data from the ENGAGE-AF trial that the risk for stroke or systemic embolization was numerically higher for edoxaban 60 mg vs warfarin (HR: 1.36; 95% CI: 0.88-2.10; *P* = 0.17).^17^ This finding should be interpreted more cautiously. First, though excellent renal clearance may result in relatively subtherapeutic drug levels and consequently less protection against stroke, the data could not be replicated in the RE-LY trial, which used the most renally cleared agent.^15^ In the RE-LY trial, efficacy in the reduction of stroke or systemic embolization in patients with eGFR ≥80 mL/min was consistent compared with patients with different ranges of eGFR (Table 2). Second, the risk for bleeding in patients with eGFR >95 mL/min was significantly lower with edoxaban 60 mg vs warfarin (HR: 0.60; 95% CI: 0.42-0.85; *P* = 0.004). Therefore, the net clinical benefit was more favorable with edoxaban.^17^ Third, the comparator (warfarin) performed unexpectedly well in the ENGAGE-AF trial, with an annual risk for stroke or systemic embolization of 0.8%.^17^ This observation has relevance when we consider the relative efficacy of a NOAC vs warfarin and may suggest that the findings described here could be due partly to excellent performance of warfarin in these patients.^17^ Fourth, an Asian analysis from South Korea demonstrated that the efficacy of edoxaban vs warfarin in reducing risk for ischemic stroke in the total population (HR: 0.67; 95% CI: 0.34-1.19), patients with CrCl > 80 to 95 mL/min (HR: 0.73; 95% CI: 0.31-1.46), and patients with CrCl > 95 mL/min (HR: 0.59; 95% CI: 0.16-1.48) was consistent, whereas the efficacy in reducing major bleeding was also consistent (HR: 0.56 [95% CI: 0.22-1.15], 0.53 [95% CI: 0.14-1.37], and 0.61 [95% CI: 0.14-1.67], respectively).^33^ Fifth, the European Medicines Agency and the regulatory authorities of 3 Asian countries (Japan, South Korea, and Taiwan) did not place any restriction on the use of edoxaban in patients with normal renal function. The consensus group therefore recommends that edoxaban can be safely used in patients with CrCl >95 mL/min.

### Consensus statements

• The burden of CKD is substantial in Asia, with some Asian countries having the highest prevalence rate of ESRD in the world.
• Patients with AF and CKD have increased risk for thromboembolism and bleeding events.
• Renal function should be monitored using the Cockcroft-Gault equation in patients on NOACs to detect worsening renal impairment and to modify NOAC doses appropriately.
• For patients with AF and mild to moderate renal impairment (CrCl 30-49 mL/min), we recommend apixaban, dabigatran 110 mg, edoxaban 30 mg, and rivaroxaban 15 mg.
• For patients with AF and severe CKD (CrCl 15-29 mL/min), apixaban 2.5 mg and edoxaban 15 mg would be preferable.
• There are no data to support the use of warfarin or NOACs in patients with AF with ESRD.
• Edoxaban can be safely used in patients with CrCl >95 mL/min.

## NOACs in Patients With Liver Disease

Liver disease is one of the leading causes of death in the Asia-Pacific region (4.6%), higher than in the United States (2.7%) and Europe (2.1%).^34^ The Asia-Pacific region contributed to 63% of global deaths due to liver disease, including liver cirrhosis, hepatocellular carcinoma, and chronic hepatitis B virus infection.^34^ Chronic hepatitis B virus infection caused more than one-half of deaths due to cirrhosis in the region, followed by alcohol consumption (20.8%), nonalcoholic fatty liver disease (12.1%), and chronic infection with hepatitis C virus (15.7%).^34^ In contrast, the presence of liver cirrhosis was independently associated with a higher risk for ischemic stroke, on the basis of a retrospective analysis of 289,559 patients with AF from the National Health Insurance Research Database in Taiwan.^35^ Considering the high prevalence of liver disease and an increasing number of patients with AF in the Asia-Pacific region, a better strategy for stroke prevention is required.

Because all NOACs depend in part on hepatic clearance, patients with elevated liver function test results, positive hepatitis viral markers, and cirrhosis were generally excluded from major NOAC trials (Table 4). Hepatic adverse events associated with NOACs were numerically lower than with warfarin in these trials (Table 4). In the ENGAGE-AF trial, 5.1% of randomized patients were found to have histories of liver diseases: elevated liver transaminase >2 times of the upper limit of normal, viral hepatitis, and liver cirrhosis.^36^ Nevertheless, a history of liver diseases did not alter the relative efficacy and safety of edoxaban compared with warfarin.^36^ Given that most patients with liver diseases were excluded from clinical trials, the safety and efficacy of NOACs in patients with impaired liver function can be obtained only from real-world evidence, including claims databases, cohort studies, and observations studies.

**Table 4:** Hepatic Clearance, Liver-Related Exclusion Criteria, and Adverse Events of NOACs

	Dabigatran	Rivaroxaban	Apixaban	Edoxaban
Hepatic clearance	20%	65%	75%	50%
Clinical trial	RE-LY^8^	ROCKET AF^9^	ARISTOTLE^11^	ENGAGE-AF^10^
Liver-related exclusion criteria				
Liver disease	Active liver disease, including active hepatitis A, B, or C	Significant liver disease (eg, acute clinical hepatitis, chronic active hepatitis, cirrhosis)		Active liver disease
ALT/AST	>2 × ULN		>2 × ULN	≥2 × ULN
ALP	>2 × ULN	>3 × ULN		≥2 × ULN
Total bilirubin			≥1.5 × ULN	≥1.5 × ULN
Viral marker	HBsAg^+^, anti-HBc IgM^+^, HCV RNA^+^			Hepatitis B antigen^+^ or hepatitis C antibody^+^
Hepatic AEs (DOAC vs VKA), OR (95% CI)^94^	0.61 (0.30-1.22)	0.94 (0.58-1.53)	0.96 (0.58-1.59)	1.50 (0.67-3.34)

Adapted with permission from Choi et al.^95^

AE = adverse event; ALP = alkaline phosphatase; ALT = alanine transaminase; AST = aspartate transaminase; DOAC = direct oral anticoagulant agent; HBc = hepatitis B core; HBsAg = hepatitis B surface antigen; HCV = hepatitis C virus; IgM = immunoglobulin M; NOAC = non-vitamin K antagonist oral anticoagulant; RNA = ribonucleic acid; ULN = upper limit of normal; VKA = vitamin K antagonist; other abbreviations as in Table 1.

**Figure 1:**
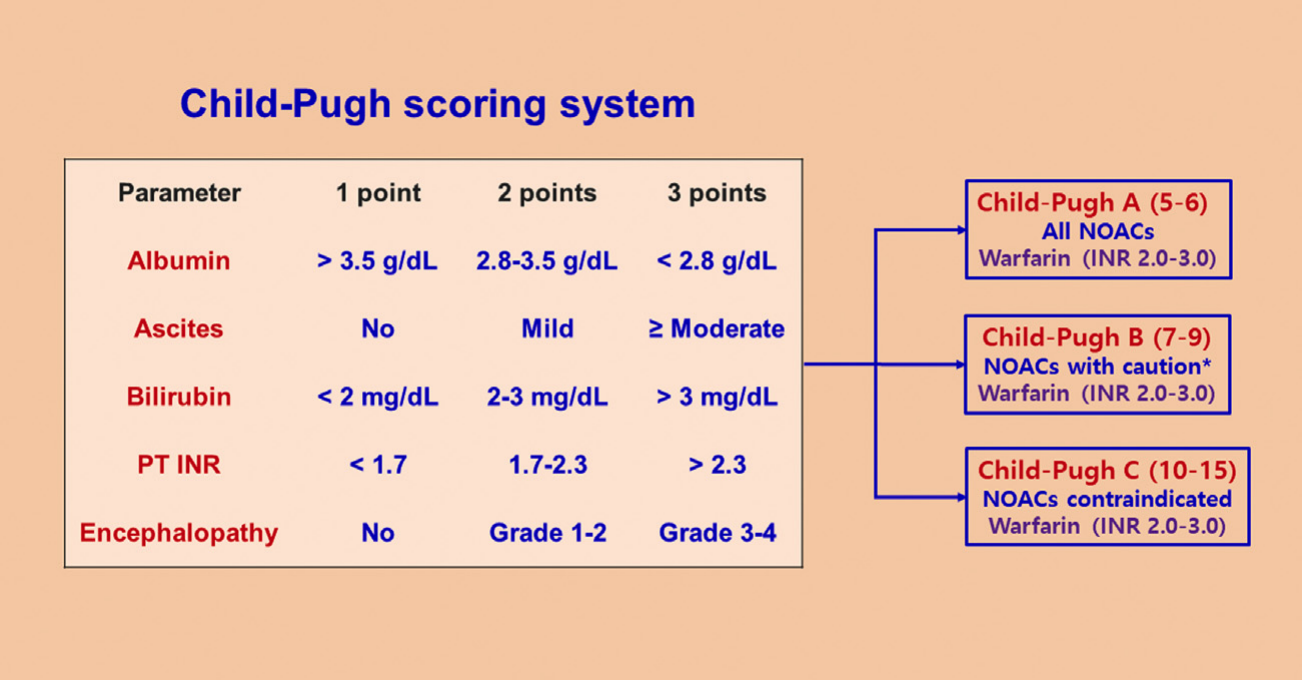
Flowchart of Management of Patients With Chronic Liver Disease∗ The Child-Pugh scoring system is used to guide the management of patients with chronic liver disease. ∗Rivaroxaban should not be used in patients with Child-Pugh B. INR = international normalized ratio; NOAC = non-vitamin K antagonist oral anticoagulant; PT = prothrombin time.

The real-world evidence for the efficacy and safety of NOACs vs VKAs in Asian patients with impaired liver function is shown in Table 5. In general, NOACs were equal to or better than VKAs in reducing stroke or systemic embolization. The majority of data suggested that NOACs were safer than VKAs in the bleeding events (major bleeding, intracranial hemorrhage, gastrointestinal bleeding) and all-cause mortality. Nevertheless, the real-world evidence should be interpreted with caution. We suggest using the Child-Pugh scoring system to grade hepatic impairment in patients with cirrhosis (Figure 1). NOACs are contraindicated in patients with Child-Pugh class C cirrhosis. For patients with Child-Pugh class B cirrhosis, dabigatran, apixaban, and edoxaban could be cautiously used,^36^ whereas rivaroxaban was not recommended as its clearance was decreased and plasma levels were increased to 2-fold in patients with Child-Pugh class B liver disease.^37^

**Table 5:** Real-World Evidence for NOACs vs VKAs in Patients With Liver Disease

Publication Year	First Author	Data Source	N	Comparison	Liver Disease	Efficacy and Safety Outcomes				
						Stroke/SEE	Major Bleeding	ICH	GIB	All-Cause Death
Asian data										
2018	Wang et al^96^ (Taiwan)	Hospital EMR	633	NOAC vs VKA	Impaired liver function^a^	0.77 (0.49-1.22), *P* = 0.271	1.31 (0.70-2.48), *P* = 0.399	NR	1.68 (0.86-3.29), *P* = 0.132	**0.64 (0.49-0.83), *P* < 0.001**
2019	Lee et al^97^ (Taiwan)	Claims database	2,428	NOAC vs VKA	Cirrhosis	NR (NOAC, 3.2%; VKA, 3.7%), *P* = 0.4296	**0.51 (0.32-0.74), *P* = 0.0003**	NR (NOAC, 1.0%; VKA, 1.6%), *P* = 0.1021	**0.51 (0.32-0.79), *P* = 0.003**	NR
			544	NOAC vs VKA	Advanced cirrhosis^b^	1.23 (0.53-2.83), *P* = 0.6303	0.51 (0.25-1.02), *P* = 0.0562	**0.17 (0.03-0.96), *P* = 0.0449**	0.69 (0.31-1.5), *P* = 0.3451	NR
2019	Lee et al^98^ (South Korea)	Claims database	37,353	NOAC vs VKA	Liver disease	**0.58 (0.49-0.62), *P* < 0.001**	**0.65 (0.58-0.74), *P* < 0.001**	**0.48 (0.39-0.58). *P* < 0.001**	**0.82 (0.62-0.95), *P* = 0.012**	**0.70 (0.64-0.77), *P* < 0.001**
			4,942	NOAC vs VKA	Significant liver disease^c^	**0.45 (0.31-0.64), *P* < 0.001**	**0.62 (0.44-0.87), *P* = 0.005**	**0.42 (0.24-0.72), *P* < 0.001**	0.76 (0.50-1.17), *P* = 0.21	0.90 (0.72-1.13), *P* = 0.35
Meta-analysis including Asian data										
2020	Dai et al^99^	6 cohorts	206,251	NOAC vs VKA	Liver disease	**0.68 (0.49-0.93)**	0.72 (0.51-1.01)	**0.49 (0.40-0.59)**	0.84 (0.51-1.36)	**0.69 (0.63-0.75)**
2021	Chen et al^100^	14 studies	20,042	NOAC vs VKA	Liver disease	0.82 (0.36-1.88)	**0.54 (0.38-0.75)**	**0.35 (0.23-0.53)**	0.72 (0.47-1.09)	0.79 (0.49-1.29)

Values are HR (95% CI), *P* value unless otherwise indicated. **Bold** denotes statistical significance.

EMR = electronic medical record; GIB = gastrointestinal bleeding; ICH = intracranial hemorrhage; other abbreviations as in Tables 1, 2, and 4.

a Defined as serum alanine transaminase or aspartate transaminase >2 times the upper limit of normal or total bilirubin 1.5 times the upper limit of normal.

b Defined as patients with cirrhosis who presented with any complications, including ascites, hepatic encephalopathy, spontaneous bacteria peritonitis, or esophageal varicose bleeding.

c Defined as subjects with liver cirrhosis, viral hepatitis, or abnormal alanine transaminase or aspartate transaminase >2 times the upper limit of normal.

The real-world evidence for NOACs vs VKAs with respect to liver injury is shown in Table 6. In general, there was no difference in the incidence hepatotoxicity between NOACs and VKAs. In fact, some studies even showed better liver outcomes among NOAC users. Given the inherent limitations of real-world evidence, the potential for hepatotoxicity of NOACs needs to be closely monitored with dedicated postmarketing surveillance.

**Table 6:** Real-World Evidence for NOACs vs VKAs on Liver Injury

Publication Year	First Author	Data Source	N	Comparison	Liver Injury Endpoints	HR (95% CI)			
						NOAC	Dabigatran	Rivaroxaban	Apixaban
Asian data									
2020	Zhao et al^101^ (Hong Kong)	Government EMR	13,698	NOAC vs VKA	ALT/AST > 3 × ULN and total bilirubin> 2 × ULN	**0.71 (0.58-0.89)**	**0.63 (0.48-0.82)**	0.72 (0.51-1.01)	1.13 (0.77-1.68)
Non-Asian data									
2017	Alonso et al^102^ (United States)	Prospective study (Medicare)	113,717	NOAC vs VKA	Liver injury hospitalization	NR	**0.57 (0.46-0.71)**	0.88 (0.75-1.03)	**0.70 (0.50-0.97)**
2018	Douros et al^103^ (Canada)	Claims database	51,887	NOAC vs VKA	Serious liver injury (hospitalization with liver injury or death due to liver injury)	Without prior liver disease: 0.99 (0.68-1.45); with prior liver disease: 0.68 (0.33-1.37)	NR	NR	NR
Meta-analysis									
2020	Dai et al^99^	Meta-analysis of 6 cohorts	206,251	NOAC vs VKA	Liver injury	**0.67 (0.56-0.80)**	**0.54 (0.44-0.67)**	**0.82 (0.70-0.96)**	**0.65 (0.45-0.95)**

**Bold** denotes statistical significance.

Abbreviations as in Tables 1, 2, 4, and 5.

### Consensus statements

• Stroke prevention is challenging in patients with AF with chronic liver disease in Asia, as Asians have the highest prevalence rate of chronic liver disease in the world.
• Patients with AF with chronic liver disease have increased risk for bleeding and thrombosis, but there is a paucity of evidence, as most patients with significant liver diseases or abnormal liver enzymes were excluded from pivotal trials.
• All NOACs can be used in patients with Child-Pugh class A cirrhosis.
• For patients with Child-Pugh class B cirrhosis, dabigatran, apixaban, and edoxaban could be cautiously used, while rivaroxaban should not be used.
• For patients with Child-Pugh class C cirrhosis, NOACs should not be used, while well-controlled VKAs may be indicated.

## NOACs in Patients With Histories of Gastrointestinal Bleeding

Gastrointestinal bleeding is a serious condition and the most common cause of major bleeding in clinical trials of NOACs.^38^ In addition, gastrointestinal bleeding is associated with significant health care costs.^39^ Patients with histories of recent gastrointestinal bleeding were excluded from 3 NOAC trials (RE-LY, ROCKET AF [Rivaroxaban Once Daily Oral Direct Factor Xa Inhibition Compared with Vitamin K Antagonism for Prevention of Stroke and Embolism Trial in Atrial Fibrillation], and ENGAGE-AF),^8^, ^9^, ^10^ whereas ARISTOTLE is the only trial that enrolled patients with histories of gastrointestinal bleeding.^11^ Except apixaban,^11^ standard doses of dabigatran, rivaroxaban, and edoxaban increased the risk for gastrointestinal bleeding compared with warfarin^40^ (Table 7). A meta-analysis of these 4 trials also demonstrated an increased risk for gastrointestinal bleeding with standard doses of NOACs compared with warfarin (HR: 1.25; 95% CI: 1.01-1.55).^41^ Interestingly, the Asian subanalyses of the RE-LY and ENGAGE-AF trials showed that dabigatran 150 mg and edoxaban 60 mg had a numerically lower risk for gastrointestinal bleeding compared with warfarin^4^^,^^7^ (Table 7). In a subsequent meta-analysis of Asian data, standard doses of NOACs had numerically lower risk than warfarin (HR: 0.79; 95% CI: 0.48-1.31) but had significantly higher risk than warfarin in non-Asians (HR: 1.44; 95% CI: 1.12-1.85).^42^ Asian subanalyses have not been reported from the ROCKET AF and ARISTOTLE trials.

**Table 7:** Risk for Gastrointestinal Bleeding of NOACs

First Author	Dabigatran	Rivaroxaban	Apixaban	Edoxaban
RCTs				
Global				
Connolly et al^8^ Patel et al^9^ Granger et al^11^ Giugliano et al^10^	150 mg vs VKA: 1.50 (1.19-1.89)	20 mg vs VKA: 1.42 (1.22-1.66)	5 mg vs VKA: 0.89 (0.70-1.15)	60 mg vs VKA: 1.23 (1.02-1.50)
Connolly et al^8^ Giugliano et al^10^	110 mg vs VKA: 1.10 (0.86-1.41)			30 mg vs VKA: 0.67 (0.53-0.83)
Asian				
Hori et al^4^ Yamashita et al^7^	150 mg vs VKA: 0.69 (0.37-1.27)	NR	NR	60 mg vs VKA: 0.91 (0.45-1.85)
Hori et al^4^ Yamashita et al^7^	110 mg vs VKA: 0.82 (0.45-1.49)			30 mg vs VKA: 0.67 (0.31-1.45)
RWE				
Abraham et al^104^ (2015) (United States)	vs VKA: 0.79 (0.61-1.03)	vs VKA: 0.93 (0.69-1.25)		
Chan et al^105^ (2016) (Taiwan)		vs dabigatran: 1.60 (1.11-2.51)		
Abraham et al^106^ (2017) (United States)		vs dabigatran: 1.20 (1.00-1.45)	vs dabigatran: 0.39 (0.27-0.58) vs rivaroxaban: 0.33 (0.22-0.49)	
Lee et al^107^ (2019) (South Korea)	vs rivaroxaban: 0.84 (0.70-0.99)		vs dabigatran: 0.78 (0.64-0.96) vs rivaroxaban: 0.68 (0.58-0.80)	vs dabigatran: 0.85 (0.66-1.08) vs rivaroxaban: 0.77 (0.62-0.94) vs apixaban: 1.19 (0.94-1.50)
Ingason et al^108^ (2021) (Iceland)		vs apixaban: 1.40 (1.01-1.94) vs dabigatran: 2.04 (1.17-3.55)		
Ray et al^43^ (2021) (Medicare)		vs apixaban: 2.09 (2.01-2.18)		
Meta-analysis including Asian data				
Gu et al^38^ (2020)	RCTs 220 mg/d vs control^a^: 1.07 (0.85-1.35) 300 mg/d vs control^a^: 0.94 (0.56-1.59) Database studies 220 mg/d vs control^a^: 0.99 (0.79-1.20) 300 mg/d vs control^a^: 1.08 (0.83-1.34)	RCTs 20 mg/d vs control^a^: 1.48 (1.22-1.80) Database studies 20 mg/d vs control^a^: 1.19 (1.08-1.30)	RCTs 10 mg/d vs control^a^: 0.70 (0.45-1.08) Database studies 10 mg/d vs control^a^: 0.65 (0.57-0.74)	RCTs 30 mg/d vs control^a^: 0.67 (0.54-0.84) 60 mg/d vs control^a^: 1.35 (0.94-1.96)

Values are HR (95% CI) unless otherwise indicated.

RCT = randomized controlled trial; RWE = real-world evidence; other abbreviations as in Tables 1 and 4.

a Antiplatelet agents or VKAs.

In the past few years, no specially designed clinical trial has been conducted to compare the risk for gastrointestinal bleeding among these four NOACs. Analysis from real-world evidence may provide some clues (Table 7). In general, rivaroxaban seems to have a higher risk for gastrointestinal bleeding compared with dabigatran, apixaban, and edoxaban. Apixaban seems to have the lowest risk for gastrointestinal bleeding, while dabigatran and edoxaban are in the middle. In the most recent systemic review and meta-analysis, the pooled rates of gastrointestinal bleeding for patients with NOACs (1.19%) vs conventional treatment (0.92%) did not differ significantly (relative risk from clinical trials: 1.09 [95% CI: 0.91-1.31]; relative risk from real-world studies: 1.02 [95% CI: 0.94-1.10]; *P* for interaction = 0.52).^38^ Rivaroxaban was the only NOAC that increased the risk for major gastrointestinal bleeding (relative risk from clinical trials: 1.39 [95% CI: 1.17-1.65]; relative risk from real-world studies: 1.14 [95% CI: 1.04-1.23]; *P* for interaction = 0.06).^38^ In a more recent study based on a U.S. Medicare database, elderly patients (≥65 years of age) with AF who were treated with rivaroxaban compared with apixaban had significantly higher risk for major ischemic or hemorrhagic events, especially gastrointestinal bleeding (HR: 2.09; 95% CI: 2.01-2.18).^43^

Several studies have shown that in patients with histories of gastrointestinal bleeding, reinitiation of NOACs was associated with lower risks for ischemic stroke, major bleeding, and gastrointestinal bleeding.^44^, ^45^, ^46^ The timing of reinitiation of NOACs after an acute episode of gastrointestinal bleeding is also under debate. In a retrospective cohort study, reinitiation of warfarin after 7 days of bleeding was not associated with increased risk for recurrent gastrointestinal bleeding but was associated with decreased risks for mortality and thromboembolism compared with resuming after 30 days of interruption.^44^ In another prospective cohort study, reinitiation of anticoagulation at discharge after gastrointestinal bleeding was associated with fewer thromboembolic events without a significantly increased risk for recurrent bleeding.^47^

It is essential to balance the risk/benefit ratio by reducing exposure to modifiable risk factors such as concomitant medications. Antiplatelet therapy,^40^ nonsteroidal anti-inflammatory drugs,^48^ and steroids are independent predictors for recurrent gastrointestinal bleeding.^49^ They should be avoided when possible. Proton pump inhibitors reduce the risk for upper gastrointestinal bleeding^50^ and should be initiated in high-risk patients who receive NOACs.

### Consensus statements

• In 4 clinical trials of NOACs vs VKAs, apixaban and dabigatran 110 mg did not increase the risk for gastrointestinal bleeding, but dabigatran 150 mg, rivaroxaban, and edoxaban 60 mg increased the risk for gastrointestinal bleeding.
• In the Asian subanalyses of the NOAC trials, dabigatran 150 mg and edoxaban 60 mg did not increase the risk for gastrointestinal bleeding.
• In real-world studies and meta-analyses, apixaban had the lowest risk for gastrointestinal bleeding. Dabigatran and edoxaban had a neutral effect, but rivaroxaban had the highest risk for gastrointestinal bleeding.
• For Asian patients with histories of gastrointestinal bleeding, rivaroxaban is the least preferred choice among NOACs.
• After active bleeding has been stopped, NOACs should be reinitiated before discharge, and proton pump inhibitors should be prescribed.
• The use of antiplatelet therapy, nonsteroidal anti-inflammatory drugs, and steroids should be minimized, especially in patients with histories of major gastrointestinal bleeding.

## NOACs in Patients With Planned Invasive Procedure, Surgery, or Ablation

Interruption of NOAC therapy in patients undergoing elective procedures is a common practice but may lead to adverse events, such as an increase in thromboembolic events by 0.3% to 0.5%, as shown in landmark NOAC trials.^51^^,^^52^ This risk needs to be weighed against an increased risk for bleeding when continuing NOACs during invasive procedures. An exhaustive list of procedures is not practical in this statement. Instead, we provide a list of common procedures with high and low risk for bleeding that is useful for periprocedural management of NOACs (Table 8).

**Table 8:** Levels of Periprocedural Bleeding Risk

Low bleeding risk (2-d risk for major bleeding 0%-2%)
• Dental extractions and other minor dental procedures, including implants and endodontic treatment
• Cataract surgery
• Noncomplex CIED insertion or electrophysiologic testing
• Noncomplex coronary or peripheral angiography
• Abdominal hernia repair
• Cholecystectomy
• Gastrointestinal endoscopy ± biopsy, enteroscopy, biliary/pancreatic stent without sphincterotomy, endosonography without fine-needle aspiration
• Hemorrhoidal surgery
• Arthroscopic surgery lasting <45 min
• Carpal tunnel repair
• Bronchoscopy with or without biopsy
• Thoracocentesis
• Central venous catheter removal
• Cutaneous and bladder/prostate/thyroid/breast/lymph node biopsies
• Hydrocele repair
• Dilatation and curettage
• Intramuscular injections (eg, vaccinations)
High bleeding risk (2-d risk for major bleeding 2%-4% or critical site bleeding)
• Multiple tooth extractions
• Any major operation of duration >45 min
• Complex invasive cardiovascular interventions, including lead extraction, (epicardial) VT ablation, chronic total occlusion PCI, etc
• Major vascular or peripheral arterial revascularization surgery
• Cardiac surgery
• Thoracic surgery
• Major abdominopelvic surgery
• Major urological surgery/biopsy
• Liver and kidney biopsy
• Prostate biopsy
• Complex endoscopy (eg, multiple/large polypectomy, ERCP with sphincterotomy)
• Major orthopedic surgery
• Laminectomy
• Spinal or epidural anesthesia, lumbar diagnostic puncture
• Neurosurgery
• Head and neck/abdominal/breast cancer surgery
• Reconstructive facial, abdominal, or limb surgery

Adapted with permission from Spyropoulos et al.^109^

CIED = cardiac implantable electronic device; ERCP = endoscopic retrograde cholangiopancreatography; PCI = percutaneous coronary intervention; VT = ventricular tachycardia.

### Periprocedural strategy

**Figure 2:**
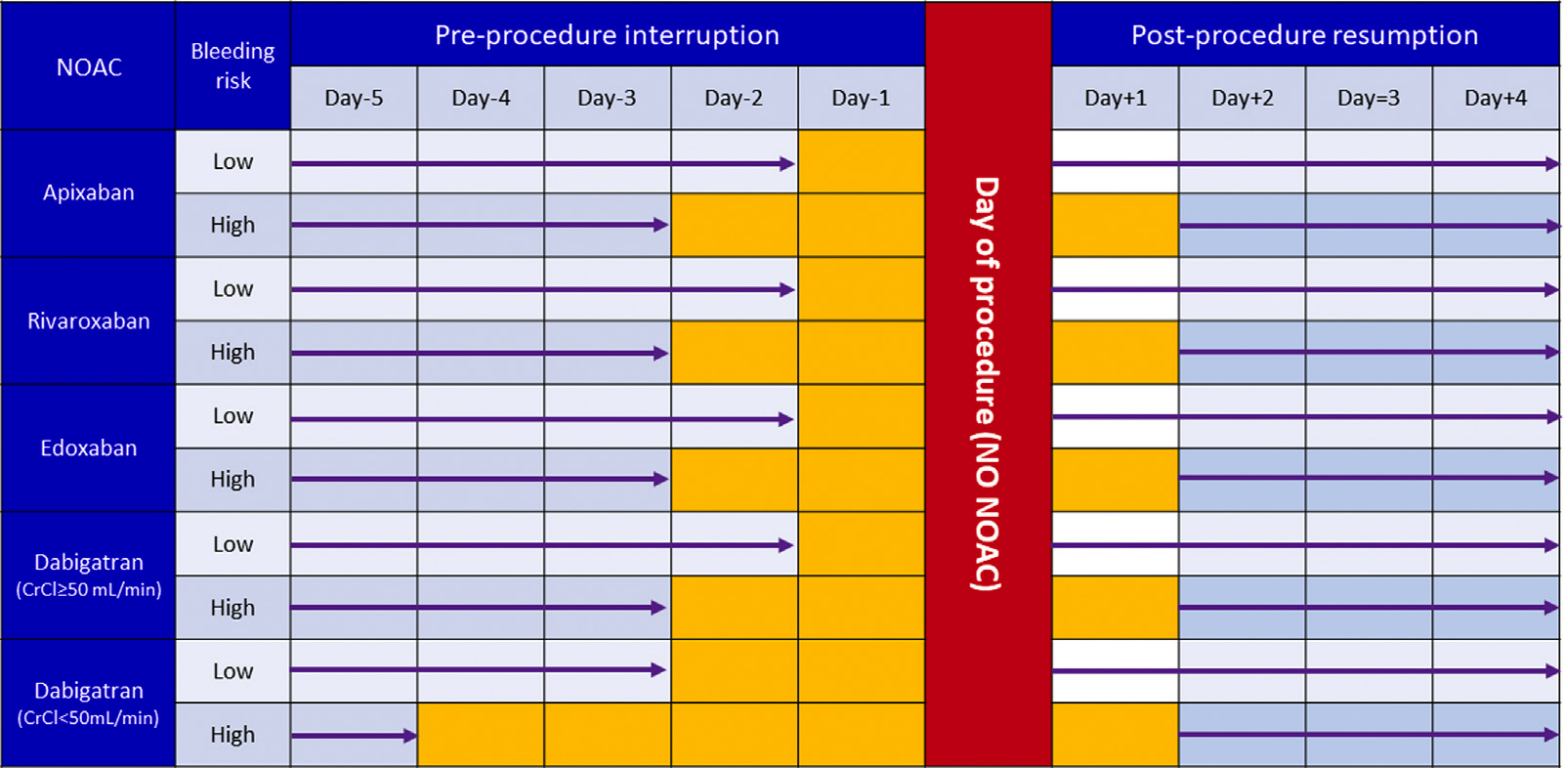
Simple Standardized Periprocedural Strategy for NOAC Use The interruption and resumption strategy for DOACs is based on the pharmacokinetic properties of NOACs, procedure-associated bleeding risk, and creatinine clearance (CrCl) levels. Refer to Table 8 to determine levels of periprocedural bleeding risk. CrCl = creatinine clearance; NOAC = non-vitamin K antagonist oral anticoagulant.

A strategy for periprocedural management of NOACs was recently provided by the PAUSE (Perioperative Anticoagulation Use for Surgery Evaluation) study.^53^ In this study, 3,007 patients with AF from 23 centers in Canada, the United States, and Europe were enrolled. Patients were scheduled for elective surgery or procedures and adhered to the NOAC therapy interruption protocol. A simple standardized perioperative NOAC therapy interruption and resumption strategy was based on NOAC pharmacokinetic properties, procedure-associated bleeding risk, and CrCl levels. The NOAC regimens were omitted for 1 day before a low–bleeding risk procedure and 2 days before a high–bleeding risk procedure. Patients using dabigatran with CrCl <50 mL/min had longer interruption intervals (2 and 4 days, respectively) to account for renal dependence of dabigatran clearance.^54^ The NOAC regimens were resumed at the first day after a low–bleeding risk procedure and at the second or third day after a high–bleeding risk procedure.^53^ With this standardized strategy, the 30-day postoperative rates of major bleeding were <2%, and the rates of stroke were <1%.^53^ On the basis of findings from the PAUSE study, we recommend the standardized periprocedural strategy in Figure 2. It may require adaptation on the basis of the individual benefit/risk ratio. Resumption of NOACs may be considered once satisfactory hemostasis has been achieved, generally 6 to 8 hours after the procedure. For procedures of low bleeding risk, it is generally safe to resume NOAC therapy the day after, or even during the evening when a procedure is performed early in the day. In cases in which bleeding risk is elevated above the risk for AF-associated thromboembolism, delaying NOAC therapy may be justified, and this decision should be made in close consultation with the proceduralist.

### Bridging is generally not recommended

For warfarin users, forgoing bridging anticoagulation was not inferior to perioperative bridging with low–molecular weight heparin, on the basis of data from the BRIDGE (Bridging Anticoagulation in Patients who Require Temporary Interruption of Warfarin Therapy for an Elective Invasive Procedure or Surgery) trial.^55^ Bridging is also not required for NOAC users, as the drugs have rapid onset and predictable pharmacokinetics. In a post hoc analysis of the ROCKET AF trial, bridging with heparin did not change thromboembolic or bleeding rates.^52^ Moreover, in the Dresden NOAC registry, perioperative bridging with heparin did not reduce cardiovascular complications, instead increased rates of major bleeding.^56^ Therefore, bridging is not routinely recommended for patients taking NOACs.

### AF catheter ablation

The most important complications associated with ablation of AF are periprocedural stroke and bleeding events, including cardiac tamponade. Systemic anticoagulation before, during, and after ablation is important in reducing the risk for periprocedural cerebrovascular events. Heparin should be administered during ablation to maintain an activated clotting time of more than 300 seconds. However, there is less consensus on preprocedural and postprocedural anticoagulation management. In the COMPARE (Role of Coumadin in Preventing Thromboembolism in Atrial Fibrillation Patients Undergoing Catheter Ablation) trial, uninterrupted VKAs during the time of ablation of AF were associated with a lower risk for periprocedural bleeding and stroke than stopping VKAs and bridging with low–molecular weight heparin^57^ (Table 9).

**Table 9:** Major RCTs and Meta-Analyses of Periprocedural Anticoagulation for Atrial Fibrillation Ablation

Publication Year	First Author	Trial Name	N	Comparison	Major Bleeding	*P* Value	Ischemic Stroke	*P* Value
Major RCTs								
2014	Di Biase et al^57^	COMPARE	1,584	Interrupted VKA vs uninterrupted VKA	0.76% vs 0.38%	0.31	3.7% vs 0.25%	<0.001
2015	Cappato et al^110^	VENTURE-AF	248	Uninterrupted rivaroxaban vs uninterrupted VKA	0% vs 0.4%	NR	0% vs 0.8%	NR
2017	Calkins et al^59^	RE-CIRCUIT	704	Uninterrupted dabigatran vs uninterrupted VKA	1.6% vs 6.9%	<0.001	0 % vs 0.3%	NR
2018	Kirchhof et al^111^	AXAFA-AFNET 5	674	Uninterrupted apixaban vs uninterrupted VKA	3.1% vs 4.4%	NR	0.6% vs 0%	NR
2019	Hohnloser et al^58^	ELIMINATE-AF	614	Uninterrupted edoxaban vs uninterrupted VKA	2.4% vs 1.7%	>0.05	0.3% vs 0%	NR
2019	Nogami et al^60^	ABRIDGE-J	442	Minimally interrupted dabigatran^a^ vs uninterrupted VKA	1.4% vs 5.0%	0.03	0% vs 0.5%	NR
Meta-analyses including Asian patients								
2018	Cardoso et al^62^	Meta-analysis	4,962	Uninterrupted NOAC vs uninterrupted VKA	0.9% vs 2% (OR: 0.50; 95% CI: 0.30-0.84)	<0.01	0.08% vs 0.16% (OR: 0.66; 95% CI: 0.19-2.30)	>0.05
2018	Ge et al^63^	Meta-analysis	12,644	Uninterrupted NOAC vs uninterrupted VKA	OR: 0.66 (95% CI: 0.45-0.96)	0.028	OR: 1.05 (95% CI: 0.44-2.52)	0.916

ABRIDGE-J = Ablation Perioperative Dabigatran in Use Envisioning in Japan; AXAFA-AFNET 5 = Apixaban During Atrial Fibrillation Catheter Ablation- Atrial Fibrillation Network 5; COMPARE = Role of Coumadin in Preventing Thromboembolism in Atrial Fibrillation Patients Undergoing Catheter Ablation; ELIMINATE-AF = Edoxaban Treatment Versus Vitamin K Antagonist in Patients With Atrial Fibrillation Undergoing Catheter Ablation; RE-CIRCUIT = Uninterrupted Dabigatran Etexilate in Comparison to Uninterrupted Warfarin in Pulmonary Vein Ablation; VENTURE-AF = A Study Exploring Two Treatment Strategies in Patients With Atrial Fibrillation Who Undergo Catheter Ablation Therapy; other abbreviations as in Tables 1, 4, and 7.

a Holding of 1 or 2 doses.

Clinical trials directly comparing each of the NOACs against uninterrupted VKAs for AF ablation, including a study from Asia, are shown in Table 9. In general, the risk for ischemic stroke was similar between NOACs and VKAs, though the actual number was quite small. The risk for major bleeding was numerically lower in NOAC group, except the ELIMINATE-AF (Edoxaban Treatment Versus Vitamin K Antagonist in Patients With Atrial Fibrillation Undergoing Catheter Ablation) trial.^58^ Interestingly, 2 trials (RE-CIRCUIT [Uninterrupted Dabigatran Etexilate in Comparison to Uninterrupted Warfarin in Pulmonary Vein Ablation] and ABRIDGE-J [Ablation Perioperative Dabigatran in Use Envisioning in Japan]) showed significant reductions of major bleeding with uninterrupted dabigatran vs uninterrupted VKAs.^59^^,^^60^ In the RE-CIRCUIT trial, only 4 patients in the dabigatran arm with major bleeding events required medical action, whereas 21 patients in the VKA arm required medical attention.^59^ Actually, dabigatran is the only NOAC that received a Class 1A indication for AF ablation in a previous consensus.^61^ Two meta-analyses that included Asian patients also concluded that uninterrupted NOACs reduced the risk for major bleeding compared with uninterrupted VKAs, while the rates of thromboembolic events were similar.^62^^,^^63^

### Consensus statements

• Perioperative bridging for surgical procedures is generally not required for patients taking NOACs.
• NOACs can be omitted for 1 day before a low–bleeding risk procedure and 2 days before a high–bleeding risk procedure.
• In patients with AF who have CrCl <50 mL/min, dabigatran should be omitted for 2 days before a low–bleeding risk procedure and 4 days before a high–bleeding risk procedure.
• NOAC regimens can be resumed on the first day after a low–bleeding risk procedure and on the second day after a high–bleeding risk procedure.
• For AF ablation, uninterrupted NOACs have similar ischemic events compared with uninterrupted warfarin.
• For AF ablation, uninterrupted NOACs, especially dabigatran, were associated with fewer bleeding complications than uninterrupted warfarin.

## NOACs in Patients With Planned Cardioversion

There have been 3 dedicated trials of NOACs in patients undergoing cardioversion (Table 10). In these trials, NOACs were compared with heparin or VKAs in patients with planned cardioversion. In general, NOACs showed similar effectiveness and safety to heparin and VKA, and in combination with transesophageal echocardiography, NOACs are useful in early cardioversion because of their rapid onset of action and easy management. A recent meta-analysis including a total of 7,588 patients from the 3 randomized studies and 4 post hoc analyses of NOAC trials also demonstrated that NOACs compared with VKAs resulted in similar risks for ischemic stroke (OR: 0.49; 95% CI: 0.20-1.19), major bleeding (OR: 0.71; 95% CI: 0.37-1.38), and mortality (OR: 0.73; 95% CI: 0.32-1.67)^64^ (Table 10). We recommend that for patients with AF or atrial flutter lasting ≥48 hours or of unknown duration, warfarin or NOAC should be used for at least 3 weeks before and 4 weeks after cardioversion. It is feasible to perform transesophageal echocardiography before cardioversion and to proceed with cardioversion if no left atrial thrombus is observed, provided that anticoagulation is achieved before transesophageal echocardiography and maintained for ≥4 weeks after cardioversion.

**Table 10:** Major RCTs and Meta-Analysis of Anticoagulation for Cardioversion

Publication Year	First Author	Trial Name	N	Comparison	Efficacy Endpoints (HR; 95% CI)	*P* Value	Safety Endpoints (HR; 95% CI)	*P* Value
Major RCTs								
2014	Cappato et al^112^	X-VeRT	1,504	Rivaroxaban vs VKA	0.51% vs 1.02% (0.50; 0.15-1.73)	NR	0.6% vs 0.8% (0.76; 0.21-2.67)	NR
2016	Goette et al^113^	ENSURE-AF	2,199	Edoxaban vs enoxaparin/VKA	<1% vs 1% (0.46; 0.12-1.43)	NR	1% vs 1% (1.48; 0.64-3.55)	NR
2018	Ezekowitz et al^114^	EMANATE	1,500	Apixaban vs heparin/VKA	0 vs 6 (0; 0-0.64)	0.015	3 vs 6 (0.49; 0.10-2.07)	0.338
Meta-analysis								
2018	Telles-Garcia et al^64^	Meta-analysis	7,588	NOACs vs VKA	(0.49; 0.20-1.19)	0.12	(0.71; 0.37-1.38)	0.32

EMANATE = Study of the Blood Thinner, Apixaban, for Patients Who Have an Abnormal Heart Rhythm (Atrial Fibrillation) and Expected to Have Treatment to Put Them Back into a Normal Heart Rhythm (Cardioversion); ENSURE-AF = Edoxaban vs. Warfarin in Subjects Undergoing Cardioversion of Nonvalvular Atrial Fibrillation; X-VeRT = Explore the Efficacy and Safety of Once-Daily Oral Rivaroxaban for the Prevention of Cardiovascular Events in Subjects With Nonvalvular Atrial Fibrillation Scheduled for Cardioversion; other abbreviation as in Tables 1, 4, and 7.

### Consensus statements

• NOACs are as effective and safe as VKAs in planned cardioversion.
• In combination with transesophageal echocardiography, NOACs are useful in early cardioversion.

## Management of Bleeding and Role of Reversal Agents

**Figure 3:**
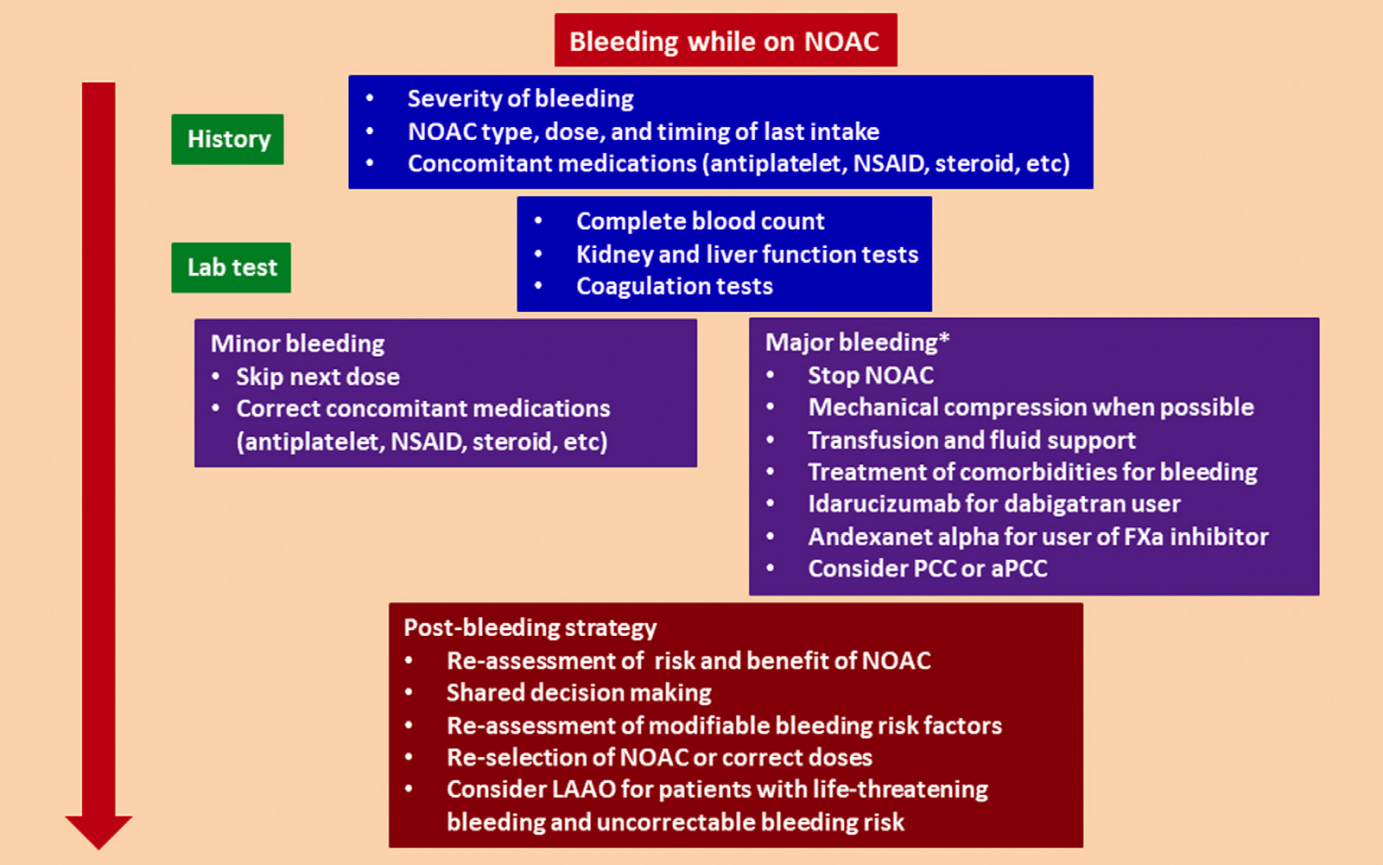
Management Strategy for Bleeding Events While Receiving NOAC Treatment When dealing with bleeding events, patients’ histories should be thoroughly checked, including types and doses of NOACs and other modifiable risk factors for bleeding, such as suboptimally treated hypertension, excess alcohol intake, concomitant antiplatelet therapy, nonsteroidal anti-inflammatory drugs (NSAIDs), steroids, and others. Furthermore, regular interdisciplinary review and shared decision making are needed. ∗Defined by International Society on Thrombosis and Hemostasis (ISTH). aPCC = activated prothrombin complex concentrate; FXa = factor Xa; LAAO = left atrial appendage occlusion; NOAC = non-vitamin K antagonist oral anticoagulant; NSAID = non-steroidal anti-inflammatory drug; PCC = prothrombin complex concentrate.

NOACs caused less intracranial and less life-threatening bleeding than VKAs in phase 3 trials,^8^, ^9^, ^10^, ^11^ especially in Asians.^42^ Moreover, patients experiencing major bleeding on NOACs had favorable outcomes compared with those on warfarin. As more patients have now been put on NOACs, the number of patients who experience bleeding episodes is increasing. Figure 3 shows the management strategy for patients with bleeding events while on NOAC treatment. When dealing with bleeding events, patients’ histories should be thoroughly checked, including type and dose of NOAC, and other modifiable risk factors for bleeding, such as suboptimally treated hypertension, excess alcohol intake, concomitant antiplatelet therapy, nonsteroidal anti-inflammatory drugs, steroids, and others. Furthermore, discussion with patients experiencing severe bleeding complications or stroke and a regular interdisciplinary review to share different subspecialty opinions as well as patient preferences is encouraged. The option of nonpharmacologic therapy such as left atrial appendage (LAA) occlusion could be provided (Figure 3).

For patients with major bleeding or life-threatening bleeding, reversal agents should be considered. Idarucizumab is a monoclonal antibody fragment and binds dabigatran with an affinity that is 350 times as high as that observed with thrombin.^65^ Its efficacy and safety have been confirmed in the RE-VERSE AD (Reversal Effects of Idarucizumab on Active Dabigatran) trial.^66^ Andexanet alfa is a recombinant modified human factor Xa decoy protein that is catalytically inactive, but it can bind factor Xa inhibitors with high affinity.^67^ Its efficacy and safety have been demonstrated in the ANNEXA-4 (A Study in Participants With Acute Major Bleeding to Evaluate the Ability of Andexanet Alfa to Reverse the Anticoagulation Effect of Direct and Indirect Oral Anticoagulants [Extension Study]) trial.^68^ Its cost and availability are major hurdles for widespread use.

### Consensus statements

• When dealing with bleeding events, patients’ histories should be thoroughly checked, including types and doses of NOACs and other modifiable risk factors for bleeding.
• For patients with major bleeding or life-threatening bleeding, reversal agents should be considered.
• For patients experiencing severe bleeding complications or stroke, a regular interdisciplinary review and shared decision making among patients and different subspecialties is needed, and nonpharmacologic therapy could be provided.

## Nonpharmacologic Management

The LAA is thought to be the predominant site of thrombus formation in patients with nonvalvular AF. A large transesophageal echocardiographic study of 1,420 patients with valvular AF or atrial flutter showed that extra-LAA thrombosis is very rare.^69^ As a nonpharmacological strategy, percutaneous or surgical closure or excision of LAA has been proposed to have the potential to reduce the risk for thromboembolism in patients with nonvalvular AF.

### LAA occlusion

Devices for trans-septal LAA occlusion include the Watchman, the Amplatzer Amulet, LAmbre, and others. The randomized controlled PROTECT AF (Watchman Left Atrial Appendage System for Embolic Protection in Patients With Atrial Fibrillation) and PREVAIL (Watchman LAA Closure Device in Patients With Atrial Fibrillation Versus Long Term Warfarin Therapy) trials have shown the noninferiority of the Watchman device to VKAs in stroke prevention in patients with nonvalvular AFs at elevated risk for stroke.^70^^,^^71^ The PRAGUE-17 (Left Atrial Appendage Closure vs. Novel Anticoagulation Agents in Atrial Fibrillation) trial showed that the Amulet or Watchman device was noninferior to NOACs for major AF-related events in patients with nonvalvular AF.^72^ However, the total patient number was only 402, and the study was underpowered to prove its effect on stroke prevention.^72^ The Amulet IDE (AMPLATZER™ Amulet™ LAA Occluder Trial) showed that the Amulet occluder was noninferior to the Watchman device for the overall safety and effectiveness but superior in the rate of LAA occlusion among patients with nonvalvular AF at increased risk for stroke.^73^ The LAmbre device had favorable clinical outcomes for stroke prevention in patients with nonvalvular AF in a prospective, multicenter, observational study in China.^74^ Procedure-related complications and device-related complications need more attention.^75^ The antithrombotic strategy after LAA occlusion has not been evaluated in large-scale randomized trials, particularly in patients with absolute contraindications to long-term oral anticoagulant therapy. For patients with nonvalvular AF with contraindications to oral anticoagulant agents, either an epicardial catheter approach (eg, Lariat system) or mini-invasive thoracoscopic LAA occlusion or exclusion may be a better alternative.^76^^,^^77^ Percutaneous LAA occlusion may be considered for patients who have absolute contraindications to NOACs or who have recurrent stroke or systemic embolization after NOACs.

There is recent interest in applying LAA occlusion in patients with ESRD.^78^ But the current data on safety and efficacy are limited to 5 small studies with a total of 84 patients.^78^ It is too early to recommend LAA occlusion as an effective alternative therapy for patients with ESRD.

### Surgical occlusion or excision of the LAA

The large randomized controlled LAAO III trial provided the evidence that LAA occlusion during cardiac surgery reduced long-term stroke risk in patients with AF, most of whom continued the ongoing antithrombotic therapy after surgery.^79^ Observational studies from Asia found that thoracoscopic LAA occlusion or excision had a reasonably low risk for thromboembolism without oral anticoagulation after the procedure.^76^^,^^77^ Therefore, mini-invasive thoracoscopic LAA intervention might be an alternative strategy for stroke prevention in Asian patients who have absolute contraindications to NOACs.

### Consensus statements

• Percutaneous LAA occlusion may be considered for patients who have absolute contraindications to NOACs or who have recurrent stroke or systemic embolization after NOACs.
• Surgical occlusion or exclusion of the LAA may have an incremental effect for stroke prevention in patients undergoing cardiac surgery.
• Mini-invasive thoracoscopic LAA occlusion might be an alternative strategy for stroke prevention in Asian patients who have absolute contraindications to NOACs.

## Artificial Intelligence for Stroke Prediction

Multimorbidity has significantly contributed to stroke complications and disability in the past 2 decades.^80^ Previously, clinical risk prediction models were based mostly on individual risk factors, and less is known about stroke prediction in diversified multimorbid conditions with roots in cardiovascular and noncardiovascular comorbid history. This becomes crucial in the presence of significant interactions of multiple comorbid conditions, their dynamic changes in risk profile over time, and their effects on clinical outcomes, including stroke. With the surge in interest in artificial intelligence and machine learning, risk prediction models can move from traditional risk tools to a new era of machine learning algorithms.^81^

Several studies have demonstrated that machine learning algorithms outperformed clinical risk factor assessment tools.^82^^,^^83^ More recently, a study based on a prospective U.S. cohort of more than 3 million patients compared 2 common clinical rules, a clinical multimorbid index, and a machine-learning approach.^84^ The machine learning algorithm yielded the highest discriminant validity, with a C index of 0.866 (95% CI: 0.856-0.876).^84^ Therefore, a machine learning approach may uncover the complex relationships of various comorbidities and their dynamic changes and facilitate automated approaches for dynamic risk stratification in the presence of multimorbidity. Innovation using machine learning and artificial intelligence approaches offers a new paradigm of “real-time” stroke risk prediction and integrated care management in the digital health era.^85^

### Consensus statement

• Machine learning facilitates dynamic risk stratification in the significant presence of multimorbidity and offers a new paradigm of “real-time” stroke risk prediction and integrated care management.

## AF-Integrated Care With Mobile Technology

Mobile technology has been used more widely in the screening and diagnosis of AF.^86^^,^^87^ In the recent mAFA II (Mobile Atrial Fibrillation Application) cluster-randomized trial using mobile technology for improving AF screening and integrated care, with a mean follow-up duration of 262 days, the rates of the composite outcomes of ischemic stroke or systemic thromboembolism, death, and rehospitalization were lower compared with usual care (1.9% vs 6.0%; HR: 0.39; 95% CI: 0.22-0.67; *P* < 0.001).^88^ Furthermore, the long-term extension cohort study of the mAFA-II trial confirmed that long-term use (≥1 year) of mobile technology in patients with AF was associated with reduced thromboembolism, bleeding events, recurrent AF or AF symptoms, and heart failure.^89^ Mobile technology–based integrated care, compared with usual care, facilitated the implementation of the ABC (Atrial Fibrillation Better Care) pathway (refer to part 1)^90^ and reduced clinical adverse events in older patients with AF and multimorbidity.^91^

### Consensus statement

• Mobile technology–based integrated care, compared with usual care, reduced clinical adverse events in patients with AF and multimorbidity.

## Selection of NOACs in Different Clinical Conditions

Although there is no head-to-head comparison among different NOACs, the effects on clinical endpoints, especially major bleeding, were different when NOACs were compared with warfarin in major clinical trials.^8^, ^9^, ^10^, ^11^ On the basis of major NOAC trials, major subgroup analyses, meta-analyses, and data from real-world evidence, we provide possible oral anticoagulant agent choices for different clinical settings (Central Illustration).^115^, ^116^, ^117^, ^118^, ^119^, ^120^, ^121^, ^122^, ^123^, ^124^, ^125^, ^126^, ^127^, ^128^, ^129^, ^130^, ^131^, ^132^, ^133^, ^134^, ^135^, ^136^, ^137^, ^138^, ^139^, ^140^, ^141^, ^142^, ^143^, ^144^, ^145^, ^146^, ^147^ The suggestions shown in the Central Illustration are not compulsory but provide possible treatment option in daily practice. Local availability, cost, and patient comorbidities should also be considered.

**Central Illustration:**
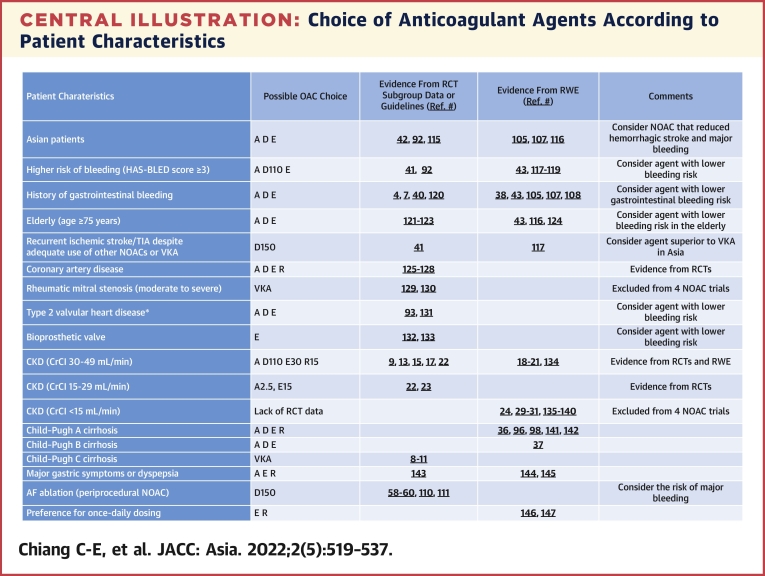
Choice of Anticoagulant Agents According to Patient Characteristics Most of the recommendations were based on randomized controlled trials or their subgroup analyses and international guidelines. A few of them were based on data from real-world evidence (RWE) or indirect evidence. Modified with permission from Lip et al.^92^ ∗Type 2 valvular heart diseases were defined previously by a European consensus,^93^ including all valvular heart diseases but excluding mechanical valves and moderate to severe mitral stenosis. A = apixaban; AF = atrial fibrillation; CKD = chronic kidney disease; CrCl = creatinine clearance; D = dabigatran; E = edoxaban; NOAC = non-vitamin K antagonist oral anticoagulant; OAC = oral anticoagulant agent; R = rivaroxaban; RCT = randomized controlled trial; Ref = reference; TIA = transient ischemic attack; VKA = vitamin K antagonist.

## Funding Support and Author Disclosures

This work was supported in part by grants from the Ministry of Health and Welfare (MOHW111-TDU-B-211-134001) and intramural grants from the Taipei Veterans General Hospital (V111C-194). Dr Chiang has received honoraria from AstraZeneca, Boehringer Ingelheim, Daiichi Sankyo, Merck Sharpe & Dohme, Novartis, Pfizer, and Sanofi. Dr Chao has received honoraria for lectures from Boehringer Ingelheim, Bayer, Pfizer, and Daiichi Sankyo. Dr Choi has received research grants or speaker fees from Bayer, Bristol Myers Squibb/Pfizer, Biosense Webster, Daiichi Sankyo, and Medtronic. Dr Krittayaphong has received honoraria from Bayer, Boehringer Ingelheim, Daiichi Sankyo, and Pfizer. Dr Li has received honoraria from Bayer and Boehringer Ingelheim. Dr Chen has received honoraria from Biosense Webster, St. Jude Medical, Medtronic, Bayer, and Boehringer Ingelheim. Dr Okumura has received honoraria from Daiichi Sankyo, Boehringer Ingelheim, Bristol Myers Squibb, Medtronic, Japan Lifeline, and Johnson & Johnson. Dr Lip is a consultant and speaker for Bristol Myers Squibb/Pfizer, Boehringer Ingelheim, and Daiichi Sankyo (no fees are received personally). All other authors have reported that they have no relationships relevant to the contents of this paper to disclose.
